# Correction: The rapid-tome, a 3D-printed microtome, and an updated hand-sectioning method for high-quality plant sectioning

**DOI:** 10.1186/s13007-024-01164-9

**Published:** 2024-04-30

**Authors:** D. J. Thomas, J. Rainbow, L. E. Bartley

**Affiliations:** 1 Department of Microbiology and Plant Biology, University of Oklahoma, Norman, OK 73019 USA; 2 Institute of Biological Chemistry, Washington State University, Pullman, WA 99164 USA

**Correction: Plant Methods (2023) 19:12** 10.1186/s13007-023-00986-3

The author of the original article [1] would like to include two supplementary files as mentioned below.

1. A new 3D print.stl file, “Rapid-Tome Sled Thick Washer Compatible_New.stl”. This file is for the sliding piece that holds the blade for the RapidTome. It makes two small adjustments to the previous “Rapid-Tome Sled.stl” file.
  The first is to slightly reduce the size of the lower part of the sled so that the sled does not make contact with the handle of the Rapid-Tome during the sectioning motion.
  The second is to make the part of the sled that holds the blade slightly thinner. This change accommodates a thicker washer that surrounds the sample opening and upon which the blade slides, as the generally available washers (such as through Amazon) seem to be a few millimeters thicker than the one which we designed the Rapid-Tome for.
2. A video that demonstrates the assembly of the Rapid-Tome after printing.

Notes: In addition, our interactions with readers and our own experience lead us to point out two of the non-printed components that seem to be particularly important for users to assemble and use their own device.

- Please note that PTFE coated blades, frequently changed out, seem to be especially important for thin sections.
- Please pay particular attention to the firmness of the foam tape piece used to hold the blade. Low-density, easily compressed foam will NOT hold the blade firmly enough. Please use high density, or “closed cell” PVC “foam” or “sponge” tape. Tape dimensions should be ¼ in. thick X 1/2 in. wide (or 6.4 mm X 13 cm). You will cut a piece that fits along the bottom of the “clamp.”

### Supplementary Information

**Additional file 1.** Rapid-Tome Sled.stl.

**Additional file 2.** RapidTome assembly video.
