# Supplementary material for: The rapid-tome, a 3D-printed microtome, and an updated hand-sectioning method for high-quality plant sectioning
Source: Plant Methods. 2023 Feb 4;19:12. doi: 10.1186/s13007-023-00986-3 (PMC9898918; doi:10.1186/s13007-023-00986-3)
Supplement: Supplementary file 1 — Additional file 1: Figure S1. Assembly of the Rapid-Tome. [file 13007_2023_986_MOESM1_ESM.docx]

**
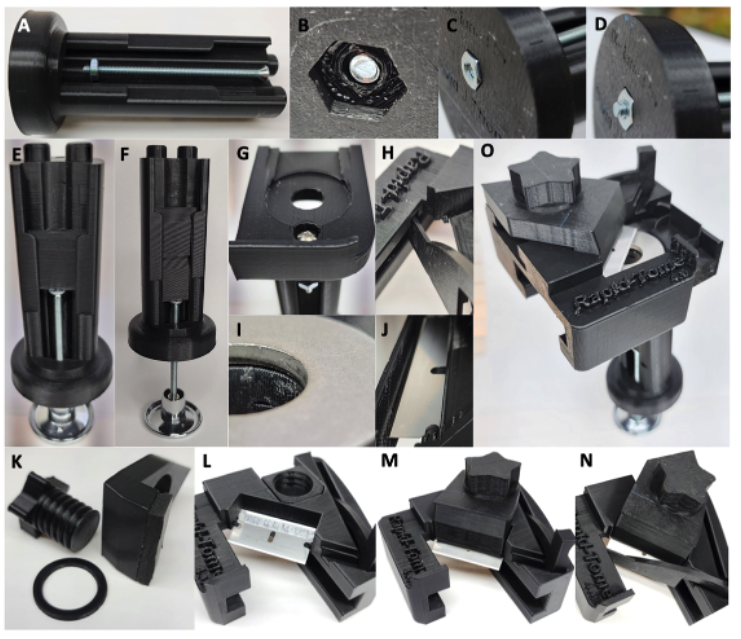
**

**Additional File 1. Assembly of the Rapid-Tome.** (A-D) The advancement bolt is installed first. (E-G) The slider is placed within the grooves of the handle before the stage is attached. (F) Two sliders can be stacked if a short sample is to be sectioned. (H-J) The washer and blade guard are attached. The blade clamp (K) is crucial and holds the blade in place (L-N). (O) Fully assembled Rapid-Tome. Sliding the sled into place over the stage against the inclined washer flexes the blade and ensures a flat cut is made. All plastic parts are 3-D printed with PLA.
