# Supplementary material for: The rapid-tome, a 3D-printed microtome, and an updated hand-sectioning method for high-quality plant sectioning
Source: Plant Methods. 2023 Feb 4;19:12. doi: 10.1186/s13007-023-00986-3 (PMC9898918; doi:10.1186/s13007-023-00986-3)
Supplement: Supplementary file 2 — Additional file 2: Figure S2. Longitudinal sections can be made utilizing a hole cut through a carrot. [file 13007_2023_986_MOESM2_ESM.docx]

**
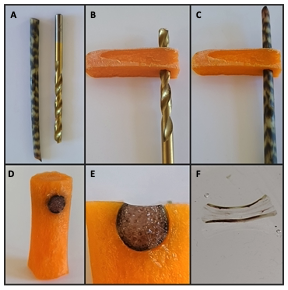
**

**Additional File 2. Longitudinal sections can be made utilizing a hole cut through a carrot.** (A) Select a drill bit that is the same diameter or slightly smaller than the *Alocasia* sp. stem segment. (B) Drill a hole through a carrot gently by hand. (C) Push the stem segment into the carrot until snug. (D) Use a razor blade to remove excess stem segment and carrot so that the carrot fits into the Rapid-Tome stage hole. (E) The carrot effectively holds the stem in place during sectioning. (F) Thin sections are possible even with fragile stem tissue.
